# Effect of SiC Addition to Al_2_O_3_ Ceramics Used in Cutting Tools

**DOI:** 10.3390/ma13225195

**Published:** 2020-11-17

**Authors:** Edwin Gevorkyan, Mirosław Rucki, Sergey Panchenko, Dmitry Sofronov, Leszek Chałko, Tomasz Mazur

**Affiliations:** 1 Department of Quality, Standardization, Certification and Manufacturing Technology, Ukraine State University of Railway Transport, 7 Feuerbach sq., 61010 Kharkiv, Ukraine; cermet-u@mail.com (E.G.); info@kart.edu.ua (S.P.); 2 Faculty of Mechanical Engineering, Kazimierz Pulaski University of Technology and Humanities in Radom, ul. Stasieckiego 54, 26-600 Radom, Poland; leszek.chalko@uthrad.pl (L.C.); tomasz.mazur@uthrad.pl (T.M.); 3 SSI “Institute for Single Crystals”, National Academy of Sciences of Ukraine, Prosp. Nauki 60, 61178 Kharkiv, Ukraine; sofronov@isc.kharkov.com

**Keywords:** SiC, ceramics, cutting tool, machining, composite

## Abstract

In this study, the effect of the addition of silicon carbide to alumina ceramics commonly used in cutting tool applications is addressed. Performance of Al_2_O_3_–SiC composite cutting inserts during the machining of hardened steels and ductile iron was compared to the results obtained for a cutting tool made out of 99 wt.% Al_2_O_3_, Al_2_O_3_–TiC, Al_2_O_3_–TiC–ZrO_2_, and Al_2_O_3_–TiN. In almost all tests, the composite with silicon carbide demonstrated better wear resistance, longer tool lifetime, and the ability to cut at higher speeds. The enhanced properties of cutting tools with SiC can be attributed to the morphology and dimensions of the inclusions in the matrix as well as to the strength of the interphase boundaries, small porosity, and lack of high inner stresses in the volume.

## 1. Introduction

Ceramic cutting tools are often applied in the machining of difficult-to-cut materials like hardened steels [1], nickel alloys [2], Inconel [3], or titanium alloys [4]. Ceramics based on alumina are important materials for this application because of their properties such as hardness retention, compressive strength, and chemical inertness at elevated temperatures, which are better than those of tungsten-cemented carbides [5]. Ruys [6] stated that these materials have an “unbeatable combination of low cost, high wear resistance, and high corrosion resistance.” Despite the advantages of Al_2_O_3_-based ceramics, they have been shown to have a lower toughness, higher probability of transverse rupture, and lower thermal conductivity when compared to other commonly used materials. These material properties contribute to a lower resistivity to mechanical and thermal shocks, which therefore limits their application in cutting tools [7]. It was found that the addition of non-oxide particles like TiC and TiN in the alumina matrix caused an increase in the thermal conductivity, the thermal shock resistance, and the hardness of the ceramics [8]. There are numerous studies on the improvement in cutting tool performance using zirconia toughened alumina (ZTA) [9,10] as well as ones with chromia addition [11], alumina reinforced with TiCN powder that contains a TiC:TiN ratio of 70:30 and an average particle size of 0.82 µm [12], or producing alumina–silver composites with Ag_2_O content from 5 to 15 wt.% and a monolithic alumina doped with MgO [13]. Other reinforcing additions such as TaC, NbC, Mo_2_C, Cr_3_C_2_ are also used [14].

Silicon carbide is widely used for the reinforcement of alumina ceramic cutting tools to improve its strength, hardness, and wear resistance [15]. Shi et al. [16] reported enhanced mechanical properties of hot-pressed Al_2_O_3_–SiC composites, and Hong et al. [17] investigated the effects of ultrasonic vibration and molding pressures on the mechanical properties and microstructures of A_2_O_3_ and Al_2_O_3_/SiC ceramic cutting tools. A significant jump in mechanical properties was attained by the inclusion of SiC whiskers, so reinforced oxides have been successfully developed for cutting tools [18]. However, it was found that ceramic fibers could be carcinogenic, and the production of whisker-reinforced materials stopped in Europe.

Silicon carbide is also added as a component to other ceramic materials for cutting tools. There are reports on Si_3_N_4_-based ceramic cutting tools with SiC addition [19,20] as well as with advanced improvements like the tailoring of phase composition and microstructure [21]. Cermet compositions based on TiCN–SiC–TiN–Cr_3_C_2_–Co–B_4_C with 15 wt.% of SiC are used for cutting tool purposes [22], and SiC for the reinforcement of diborides HfB_2_, ZrB_2_, and TiB_2_ ceramic cutting tools [23].

The present study is dedicated to the effect of SiC on the performance of cutting tools made out of alumina ceramic composites compared to other additions such as TiC, TiN, and ZrO_2_. In particular, the studies focused on microdispersed alumina with the addition of 15 wt.% SiC nanopowder, sintered with the modified hot-pressing device with activation by directly applied current.

## 2. Materials and Methods

The initial studies proved that products from the proposed material based on micro powder Al_2_O_3_ with an additive of nano-powder SiC can be used for the manufacture of heat-stressed parts operating at temperatures up to 2000 °C in conditions that require high strength, hardness, and oxidation resistance as well as in conditions of thermal shock as experienced by the cutting tools [24]. After thorough investigations on the Al_2_O_3_–SiC composites of different SiC proportions from 0 up to 50 wt.%, it was found that the materials with 15 wt.% SiC reached their density close to 99% and performed the highest bending strength of 600 MPa [25]. Aluminum oxide performed unique abrasive properties even in the form of sintered powder after hot-pressing. Thus, for the present study, the material denoted As15-6 was chosen for the tests as a material for the cutting tool designed for precise machining of hardened steels. Its composition consisted of microdispersed alumina produced by Zaporozhsky Abrasivny Combinat (Zaporizha, Ukraine) with the addition of 15 wt.% SiC nanopowder produced by Saint-Gobain (Courbevoie, France).

To fabricate the cutting inserts, a powder metallurgy method was chosen [26]. The sintering process was performed using the modified hot-pressing device with activation by directly applied current [27] at temperature *T_sint_* = 1600 °C under the axial pressure *P* = 35 MPa during the sintering time *τ_sint_* = 3 min. These parameters ensured that the bulk material obtained a fine structure and high density.

**Figure 1:**
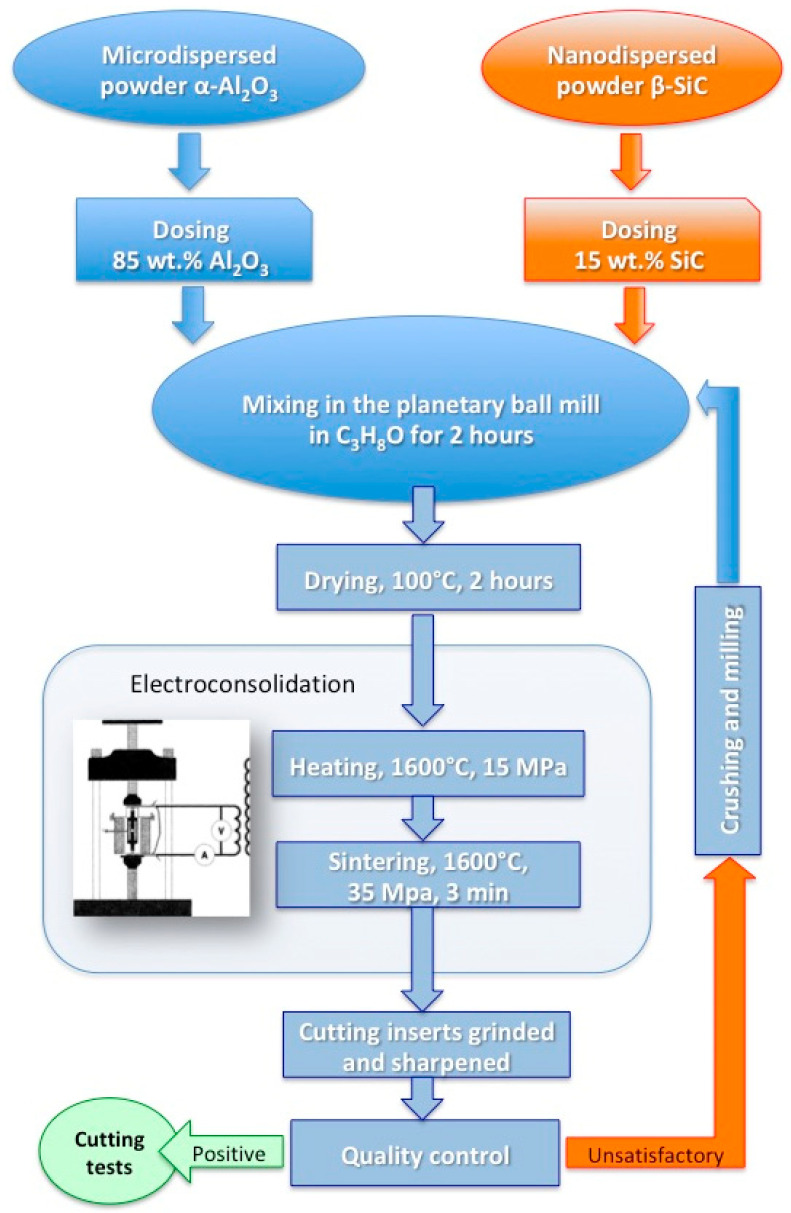
Preparation of the As15-6 cutting inserts for the tests.

Initial powders of aluminum oxide and silicon carbide were mixed in ceramic drums in an isopropyl alcohol environment using planetary ball mill for 2 h. Next, the powders were dried in a vacuum drying camera and then forced through a sieve. The mixture at certain doses was placed in the graphite mold and underwent electrically activated hot pressing. The entire procedure is presented in the block diagram in Figure 1.

Hot pressing in vacuum enabled us to obtain a composite with finely dispersed structures and high purity of the grain boundaries, which resulted in enhanced mechanical properties of the bulk material. A short sintering time prevented excessive grain growth. The structure of the obtained cutting inserts consisted of the aluminum oxide matrix with randomly scattered grains of SiC. The latter had dimensions up to 10 times larger than that of the SiC initial powder, which meant an agglomeration process between its nanoparticles throughout the preparation and sintering procedures.

After sintering, the ready cutting tool inserts were ground with diamond grinding discs 6A2-100/80 of grit size 100/80, produced by the Institute of Superhard Materials (Kharkov, Ukraine), with the application of an organic binding agent. The cutting velocity of grinding was *v* = 25–30 m/s and the cross feed was *S_c_* = 0.02 mm per double pass.

The cutting tests were performed with a universal lathe 16K20 produced by Moscow Machine Tools Factory (Moscow, Russia) using a Sandvik-TL-120406 Tool Holder (Sandvik Coromant, Fair Lawn, NJ, USA). The machined materials were hardened steels and ductile cast iron as specified in the Table 1. In the tests, the cast iron BЧ80 (Russian nomenclature) was machined, which has its closest analogue in UE nomenclature EN-GJS-800-2 or EN-JS1080. These materials usually produce long, stringy swarf, making conditions of machining more difficult. In particular, the temperature in the cutting zone increases, and adhesive and abrasive wear processes are intensified, especially at higher cutting speeds.

Comparative cutting tests (turning) were conducted in similar conditions for each material: velocity *v_c_* = 100–300 m/min, feed rate *f* = 0.085–0.3 mm/rev, and cutting depth *a_p_* = 0.1–0.5 mm.

In the tests, commercially available cutting inserts were used, as described in Table 2. Based on our own experience, it was assumed that the above-mentioned materials were commonly machined with the cutting inserts BO-13, BOK-60, BOK-71, and OHT-20 used in the experiments. The turning operation was continued until the surface of the cutting insert became worn. The critical wear criterion was worn flank width *h =* 0.4 mm.

Characteristics of the insert material were measured with the following methods. The fracture toughness was measured along the microhardness *HV* test on the basis of the size of the indentation diagonals and length of cracks left by the indenter in the form of a square-based pyramid with an angle α = 136°. The tests were performed using the DM8 device produced by Affri (Induno Olona, Italy). Microhardness was then calculated from the following Equation:

(1) $$HV=\frac{kP}{{\left(2a\right)}^{2}}$$

where *k* is a coefficient dependent on indenter shape, here *k =* 1.854; *P* is the load in kg; and *a* is the mean value of two measured diagonals in μm. Then, the fracture toughness *K_Ic_* was calculated to assess the crack resistance of the material, as follows:

(2) $$K_{Ic}=0.016{\left(\frac{l}{a}\right)}^{-0.5}{\left(\frac{HV}{E\Phi }\right)}^{0.4}\frac{HVa^{0.5}}{\Phi }$$

where *E* is elasticity modulus [GPa]; *HV* is microhardness [GPa]; Φ is a constant, here Φ = 3; and *l* is the crack length from the indentation angle. The elasticity modulus was determined using the standard methodology described in ISO 3312-75 with the measurement error no larger than ±0.2%.

In the study, a series of cutting inserts were prepared out of the As15-6 powder mixture, as described above. Next, the cutting tests were performed using these inserts and the ones chosen for comparative analysis. Finally, the structure and properties of the As15-6 material were examined to find out the effect of the SiC addition.

## 3. Results and Discussion

From the perspective of the cutting tool application, it is crucial to obtain a strong material with high wear resistance. In the case of the As15-6 composite, its fracture toughness was *K_Ic_* = 6.5 ± 0.2 MPa·m^½^, the flexural strength was *σ_f_* = 600 ± 10 Mpa, and the hardness *HV*(15) = 20.3 ± 0.2 GPa. The characteristics were close to the ones obtained for the widely applied commercial material BOK-71, which consists of the Al_2_O_3_–TiC composite with the addition of ZrO_2_. However, our material As15-6 had outstanding thermal conductivity (*κ* = 35 W/m·K), 1.5 times better than that of the other materials presented in Table 2. This characteristic is of high importance for cutting tools.

**Figure 2:**
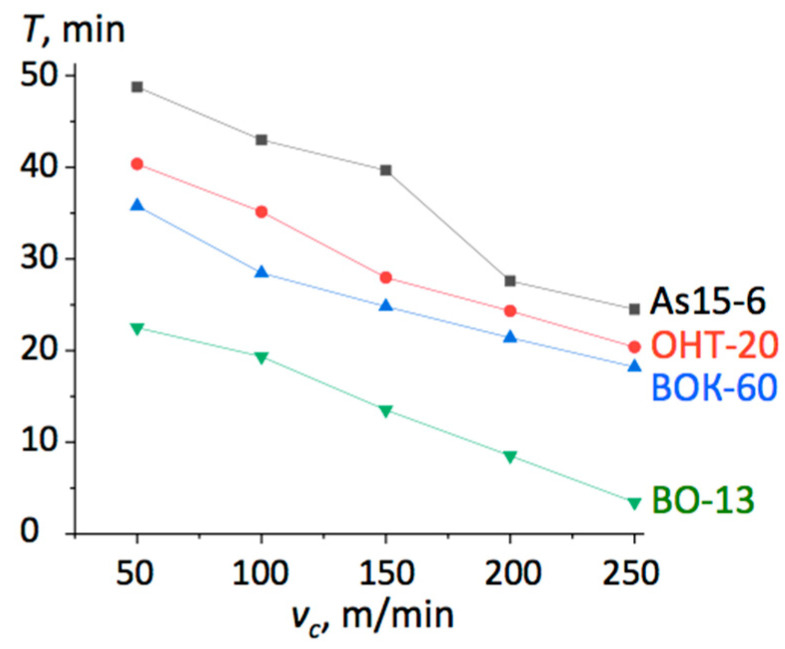
Lifetime *τ_l_* of different cutting edges versus cutting velocity during the machining of steel 100Cr6 (HRC 58-60) with *f* = 0.15 mm/rev and *a_p_* = 0.2 mm.

The results of the cutting tests are presented in Figure 2 in the form of graphs of tool lifetime dependent on the cutting velocity. The lifetime *τ_l_* was considered to be the time when the flank wear reached the value *h_з_* = 0.3 mm.

When cutting the hardened steels 59CrV7 and 100Cr6 with *v_c_* = 120 m/min, *f* = 0.1 mm/rev, and *a_p_* = 0.2 mm, the lifetime of the tested composite As15-6 was ca. 65 min, comparable to that of the BOK-71 inserts. In both cases, the machined surface roughness was *Ra* = 0.70. However, with a higher feed of *f* = 0.25 mm/rev, the lifetime of the As15-6 reduced by 50%, while that of BOK-71 reduced by 75%. Moreover, the BOK-60 inserts chipped off in the first few seconds of cutting. When the cutting velocity was increased up to *v_c_* = 200 m/min, the lifetime of As15-6 was *τ_l_* = 15 min, five times longer than that of BOK-71.

**Figure 3:**
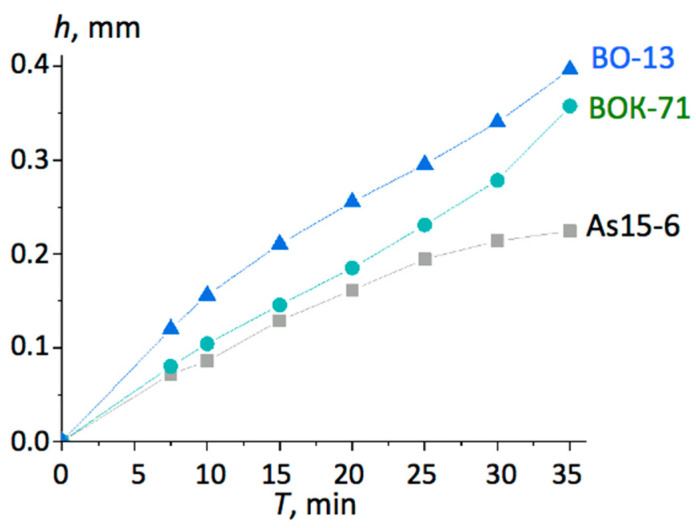
The tool wear curves for C45 steel (HRC 40-45) machined with *v_c_* = 200 m/min, *f* = 0.085 mm/rev, and *a_p_* = 0.2 mm for the tested inserts.

Figure 3 presents the tool wear curves for the three different cutting tool materials.

It can be noted from Figure 3 that commercially available inserts made out of alumina (BO-13) and alumina with carbide (BOK-71) performed intensified wear after ca. 20 min of cutting, while the As15-6 composite showed the opposite trend, slowing down the wear process. Most likely, this demonstrated that the machining conditions were optimal for this new material, since one of the goals of the research on cutting tool material was to improve its thermal and physical properties, thus enabling an increase in cutting speed. Thermal diffusivity of the new composite As15-6 was *a* = 9.4·10^−6^ m^2^/s, which was at least 50% higher than that of other similar alumina-based ceramics. This contributed to the accelerated heat removal from the cutting area and thus protected the edge from thermal shocks that are highly destructive for ceramics. This may be the main mechanism behind the trend seen in Figure 3.

**Figure 4:**
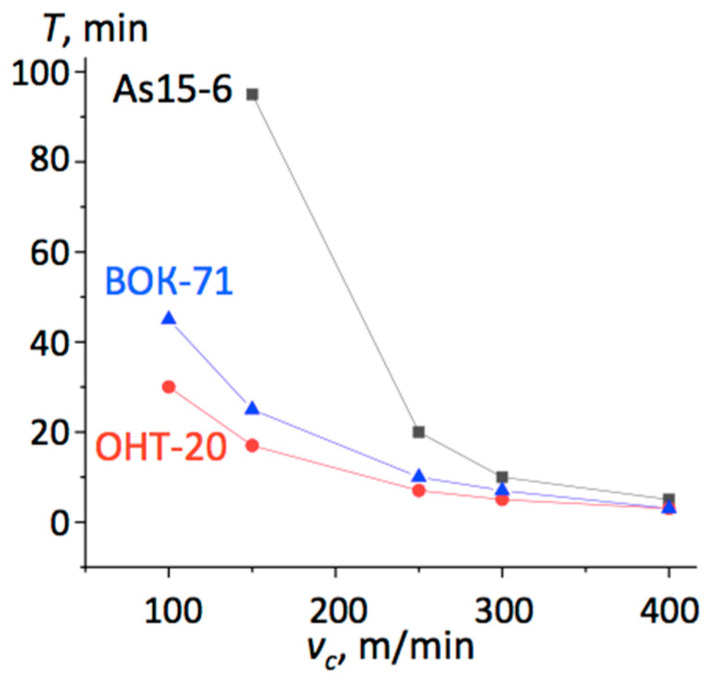
Lifetime *τ_l_* of the different cutting edges versus cutting velocity during the machining of the ductile iron BЧ80 with *f* = 0.2 mm/rev and *a_p_* = 0.5 mm.

**Figure 5:**
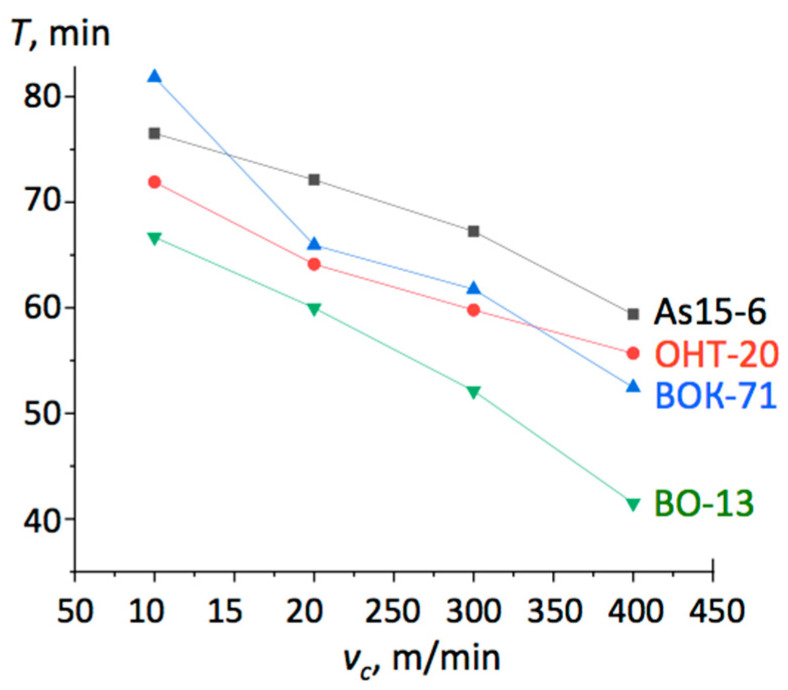
Lifetime *τ_l_* of the different cutting edges versus cutting velocity during the machining of C45 steel (40-45 HRC) with *f* = 0.085 mm/rev and *a_p_* = 0.2 mm.

Examples of the lifetime *τ_l_* curves plotted for cutting edges from different materials, shown in Figure 4 and Figure 5, demonstrated that in most cases, the As15-6 composite performed the best. Especially spectacular was the result for cast iron cutting at lower speed *v_c_* = 150 m/min, where it worked three times longer than the cutting inserts fabricated from other materials. Even though its lifetime was also the longest at higher cutting speeds, the differences above *v_c_* = 300 m/min were small, as seen in Figure 4. In the case of the C45 steel hardened up to 40-45 HRC, as demonstrated in Figure 5, the As15-6 cutting insert performed better than others at higher speeds, namely, *v_c_* = 200 m/min and more.

During the wear process of a cutting tool, pits appeared in the rake surface and chamfers in the flank. It was found that the wear process of the As16-5 ceramic could be described in general terms of cutting tool wear [28] and from the perspective of the dislocation-based model [29]. After grinding with diamonds, the grains of the oxide, nitride, and carbide phases in ceramics showed a certain dislocation density. As a result of outer friction during machining, the dislocation density in the grains increases. Analysis of the microphotographs of worn areas proved that As15-6 ceramics were subjected to the microdestruction of grains as the dislocation density increased above critical values, and then the grains were cyclically loosened and torn out.

**Figure 6:**
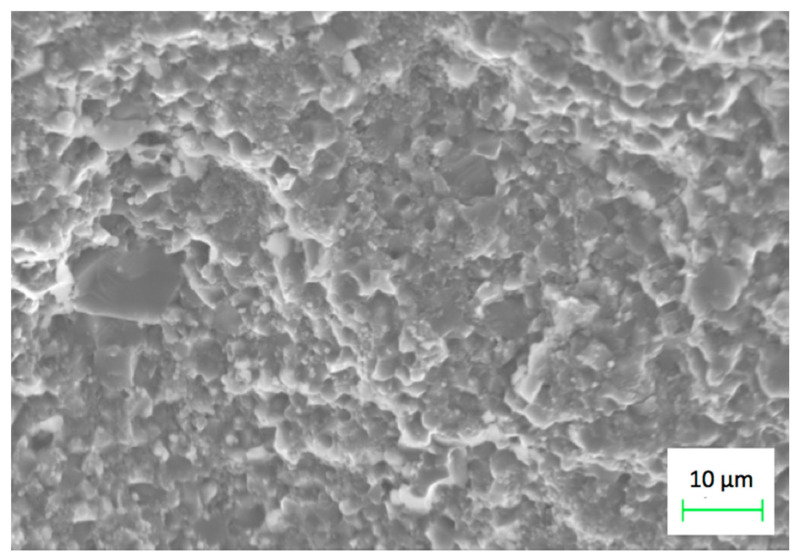
Fractogram of the As15-6 insert after the cutting test.

This hypothesis was confirmed by the fractographic analysis. It can be seen in the scanning electron microscopy (SEM) image in Figure 6 that the intercrystalline destruction mechanism prevailed, while the cleavage of larger grains rather had a transcrystalline nature. Material creep in the outer layers and appearance of pores during the wear process led to the appearance of microcracks between the grains, and subsequent chipping of the cutting edge. In the case of the As15-6 composite, its increased wear resistance could be attributed to its high dispersity and the presence of misfit stresses around SiC inclusions. It is known that crack propagation around the included particle requires more energy, and the misfit stresses usually cause crack arrest and crack deflection [30].

## 4. SiC Effect on the Structure and Properties of the As15-6 Composite

**Figure 7:**
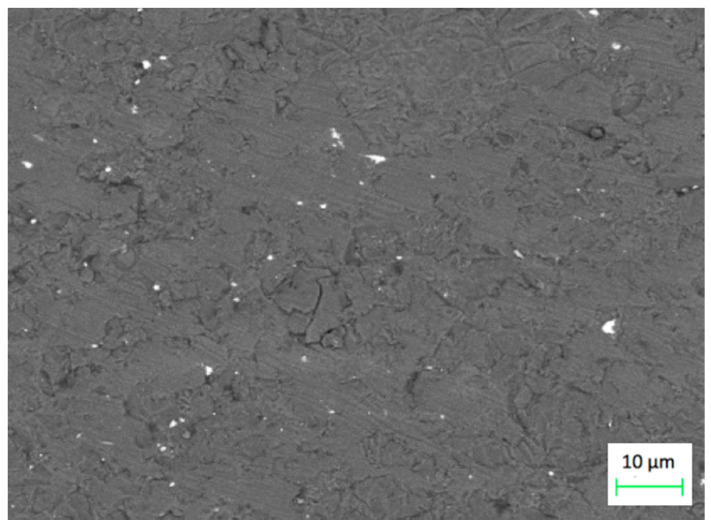
Surface structure of the As15-6 composite after diamond grinding.

The outer layer of the ceramic composite As15-6 after diamond grinding was analyzed at a depth of 2–5 μm, which was found to be integral, without high inner stresses in the volume. Porosity did not exceed 1%, and the pores had a rather regular round form, as shown in Figure 7, which additionally contributed to decreased inner stresses in the volume.

Structural SEM analysis of the compared ceramic cutting tool materials BO-13, BOK-60, BOK-71, and OHT-20 demonstrated that the materials of their class could be treated as dispersion-strengthened ceramics [31]. It is known that the amount of crack deflection and effective strengthening of ceramics depends on the shape of the included particles, and spherical inclusions cause a definite improvement in the mechanical properties [30]. In the tool materials compared in this study, the alumina matrix was strengthened with the respective dispersed particles TiC, ZrO_2_, or TiN, as shown in Table 2, while in As15-6, the included particles were SiC. In the BO-13 material, Al_2_O_3_ grains reach size of 2–5 μm. In the oxide-carbide materials BOK-60 and BOK-71, the average grain size was also 2–5 μm, but with TiC inclusions of 0.2–0.7 μm. In the As15-6 composite, the strengthening SiC particle size was much smaller, ca. 50–100 nm. Additionally, the content of strengthening particles was different. For instance, in BOK-60, there was 40 wt.% of TiC, while in As15-6, there was only 15 wt.% of SiC. These features had an effect on the physical and mechanical properties shown in Table 2. Especially important was the highest hardness of As15-6, which was HRA = 94, similar to BOK-60, the highest bending strength *σ_bnd_* = 0.85 GPa, and the highest fracture toughness *K_Ic_* = 6.5 MPa·m^1/2^.

Dispersion strengthening is largely determined by the morphology and dimensions of the inclusions in the matrix and their proportion as well as by the strength of the interphase boundaries. The latter is dependent on the ratio of the elastic modulus of inclusions to that of the matrix, their thermal expansion coefficients (CTEs, denoted *α*), and the solubility of the strengthening phase in the matrix. The elastic modulus of the included particles should be larger than that of the matrix, so that the stress concentration in the boundaries may be higher. Respective values are presented in Table 3 to demonstrate the differences between the tested cutting tools.

It can be seen from Table 3 that the silicon carbide generated a higher stress concentration in the phase boundaries than the titanium carbide or nitride. Moreover, SiC grains had a higher dispersity and, thus, they were more coherent with the matrix. These parameters contributed to the strength of the interphase boundaries and can explain the enhanced strength of As15-6 tools when cutting hardened steels.

In order to estimate the possibility of a reaction between the machined material and structural components of the cutting tool ceramics, thermodynamic potential calculations may be applied. The measurements of temperature in the cutting area proved that it could reach the values of the iron phase transition Fe_α_ → Fe_γ_ in the thin surface layers. High pressure initiates dissociation of cementite. As a result, the main elements able to diffuse into the surface layers of the ceramic are ionized iron and carbon present in austenite. The reaction can be described as follows:

Al_2_O_3_ + 2Fe → 2Al + Fe_2_O_3_ (3)

However, the calculations demonstrated that the thermodynamic potential of Reaction (3) was positive in the wide temperature range, hence, Reaction (3) is energetically disadvantageous [32].

On the other hand, reactions with carbon are possible only at the temperatures above 1800 °C:

2Al_2_O_3_ + 9C → Al_4_C_3_ + 6CO (4)

Such temperatures are far beyond the ones that may appear in the cutting area [33]. The actual working temperature of 1100–1300 °C makes possible the reactions with silicon, manganese, and calcium present in the steel as well as with their oxides. X-ray spectral analysis, however, did not reveal carbides and pure Fe and Al either in the chips or in the machined material, which should have been expected as a result of the restoration of oxides. Nevertheless, in the layers of machined material adhered to the tool surfaces, there were oxides of FeO and Fe_2_O_3_.

Thus, a comparative analysis of different ceramic cutting tool materials led to the conclusion that the enhanced wear resistance of the oxide–carbide ceramic cutting tools, especially of the new composite As15-6, was mainly due to the fine grain structure. Additionally, substructural and dispersal strengthening mechanisms were achieved. In the pure Al_2_O_3_ ceramics, there were no dislocations in the grains, so the grains were unable to store the deformation energy. As a result, microscale cleavage of the surface layer grains took place. Hence, fragile destruction may be prevented by a decrease in the matrix grains. This effect can be reached due to the addition of carbides TiC or SiC, which was demonstrated in our paper.

## 5. Conclusions

Theoretical expectations on the dispersion strengthening of alumina ceramic cutting tools were experimentally confirmed. This was achieved with the addition of nanodispersed silicon carbide to microdispersed Al_2_O_3_. Even though SiC formed agglomerates much larger than the initial particles, their dimensions remained below 100 nm and the dominant shape was spherical. These features contributed to the increased wear resistance and prolonged lifetime of the cutting tools.

The cutting tests with hardened steels demonstrated that the proposed material enabled an increase in cutting speed. Both the precise and semi-precise machining cutting speed could be two times higher than that of the cutting inserts of commercially available ceramics with TiC additions. Moreover, the As15-6 tool wear curve for the machining of C45 steel showed a decreasing trend of wear with time, unlike other materials. The lifetime of the As15-6 cutting tools was longer than that of the other ceramics, and in the case of cast iron machining, it was three times longer than the others.

Further improvement in the performance of As15-6 cutting tools can be reached through the following operations: optimization of the diamond grinding process and its parameters, polishing, spraying the defective surface layer, or damping of the cutting tool in the normal direction, where the latter can be attained with thicker inserts. After machining of the hardened steel with the test inserts, the obtained surface quality was enhanced and sometimes similar to the one after grinding. Possible elimination of subsequent grinding operations after turning indicate additional possible savings from the industrial application of the new ceramic material with 15 wt.% SiC addition.

## 6. Patents

Gevorkyan E.S., Azarenkov M.O., Litovchenko S.V., Chishkala V.O., Timofeeva L.A., Melnyk O.M., Gutsalenko Yu.G. Device for hot-pressing of powders by direct transmission of electric current. Patent No. 72841, Ukraine (in Ukrainian).

Vovk R.V., Gevorkyan E.S., Timofeeva L.A., Panchenko S.V., Chishkala V.O., Litovchenko S.V., Kislitsa M.V. Composite material of high mechanical and thermal characteristics with silicon carbide addition. Patent No. 18903, Ukraine (in Ukrainian).

## Figures and Tables

**Table 1:** Machined materials used in experiments.

Machined Material	Hardness, HRC
C45	40–45
59CrV7	58–60
100Cr6	58–60
BЧ80	38–40

**Table 2:** Material of the cutting tools used in experiments.

Denotion	Composition	Fabrication Method	HRA	*ρ*, g/cm^3^	*σ_bnd_*, GPa	*K_IC_*, MPa·m^1/2^	*κ*, W/m·K	Grain Size, μm
BO-13	99 wt.% Al_2_O_3_	Sintering	92	3.95	0.50	3.5	20	3–4
BOK-60	Al_2_O_3_-TiC	Hot pressing	94	4.30	0.60	4.2	22	2–3
BOK-71	Al_2_O_3_-TiC-ZrO_2_	Hot pressing	92–93	4.52	0.65	5.6–6.0	22	2–3
OHT-20	Al_2_O_3_-TiN	Hot pressing	90–92	4.39	0.64	4.5	-	2
As15-6	Al_2_O_3_-SiC	Hot pressing	94	3.83	0.85	6.5	35	1–2

**Table 3:** Ratios of *E* and CTEs for the different inclusions in the Al_2_O_3_ matrix.

Parameter	Ratio Inclusion/Matrix	$\frac{TiN}{Al_{2}O_{3}}$	$\frac{TiC}{Al_{2}O_{3}}$	$\frac{SiC}{Al_{2}O_{3}}$
Elasticity modulus	*E*_1_/*E*_2_	0.7	1.3	0.9
CTE [31]	α_1_/α_2_	1.2	1.0	2.0
